# Covalent Label Transfer between Peroxisomal Importomer Components Reveals Export-driven Import Interactions*

**DOI:** 10.1074/jbc.M115.686501

**Published:** 2015-11-13

**Authors:** Moninder S. Bhogal, Thomas Lanyon-Hogg, Katherine A. Johnston, Stuart L. Warriner, Alison Baker

**Affiliations:** From the ‡ Centre for Plant Sciences and School of Molecular and Cellular Biology and; § School of Chemistry and Astbury Centre, University of Leeds, Leeds LS2 9JT, United Kingdom

**Keywords:** Arabidopsis, peroxisome, protein chemical modification, protein import, protein-protein interaction, PEX14, PEX5, biotinylation

## Abstract

Peroxisomes are vital metabolic organelles found in almost all eukaryotic organisms, and they rely exclusively on import of their matrix protein content from the cytosol. *In vitro* import of proteins into isolated peroxisomal fractions has provided a wealth of knowledge on the import process. However, the common method of protease protection garnered no information on the import of an N-terminally truncated PEX5 (PEX5C) receptor construct or peroxisomal malate dehydrogenase 1 (pMDH1) cargo protein into sunflower peroxisomes because of high degrees of protease susceptibility or resistance, respectively. Here we present a means for analysis of *in vitro* import through a covalent biotin label transfer and employ this method to the import of PEX5C. Label transfer demonstrates that the PEX5C construct is monomeric under the conditions of the import assay. This technique was capable of identifying the PEX5-PEX14 interaction as the first interaction of the import process through competition experiments. Labeling of the peroxisomal protein import machinery by PEX5C demonstrated that this interaction was independent of added cargo protein, and, strikingly, the interaction between PEX5C and the import machinery was shown to be ATP-dependent. These important mechanistic insights highlight the power of label transfer in studying interactions, rather than proteins, of interest and demonstrate that this technique should be applied to future studies of peroxisomal *in vitro* import.

## Introduction

Peroxisomes are present in almost all eukaryotes, where they perform a diverse range of metabolic functions. Peroxisomes can arise *de novo* from the endoplasmic reticulum, but because they possess no DNA or protein synthesis machinery, they rely on the post-translational import of their matrix protein content from the cytosol (1). Import is performed by a protein machinery termed the “importomer,” which contains several components that are conserved across different species (2). One of the most unusual features of the importomer is its ability to import folded proteins, and even oligomeric protein complexes, across the peroxisomal membrane without compromising the integrity of the organelle.

Two targeting signals direct proteins to the peroxisomal matrix. Peroxisomal targeting signal type 1 (PTS1) is the major mode of targeting in all species and consists of a tripeptide motif at the C terminus of the matrix protein that generally conforms to the consensus sequence of [S/A/C]-[K/R/H]-[L/M]-COOH (3). Recent proteomic, *in vitro* binding, and *in vivo* targeting analysis in plants has led to the identification of further expanded PTS1 consensus sequences and highlighted the ability of lower-affinity PTS1 motifs to import *in vivo* (4, 5). Peroxisomal targeting signal type 2 (PTS2) is found in a smaller number of matrix proteins than the PTS1 signal, but plants possess a larger number of PTS2 proteins than other organisms (4). The PTS2 sequence is a nonapeptide located at or near the N terminus of the matrix protein and has the consensus sequence [L/V/I]-*X*_5_-[H/Q]-[L/A] (where *X* denotes any amino acid) (6).

The PTS1 sequence is recognized by cytosolic receptor PEX5 (7, 8), where the PTS1 tripeptide binds to a series of tetratricopeptide repeat motifs in the C-terminal of PEX5 (9). The PTS2 sequence is recognized by the cytosolic receptor PEX7, which binds its PTS2 cargo in concert with an ancillary co-receptor protein (10). In plants, this co-receptor is PEX5. In mammals, it is a longer splice variant of PEX5 termed PEX5L, and in yeast, this role is performed by PEX18, PEX20, or PEX21 (11). Recent structural studies from yeast have shown PEX7 possesses a seven-bladed β propeller structure, with co-receptor interaction forming a hydrophobic pocket to bind the PTS2 cargo (12).

This receptor-cargo complex subsequently docks at the peroxisomal membrane by interacting with the peroxisomal membrane proteins PEX13 and PEX14, termed the docking complex. PEX5 is also capable of functioning as a membrane protein by inserting into the peroxisomal membrane to form a ligand-gated dynamic membrane pore that opens in response to the receptor-cargo complex (13). This dynamic pore provides a rationale for how bulky cargoes may translocate across the membrane without compromising membrane integrity, but it does not reveal the actual mechanism of receptor-cargo translocation.

Two models predominate for translocation of the receptor-cargo complex. The simple shuttle model proposes that the receptor is only partially exposed to the peroxisomal lumen, whereas the extended shuttle model proposes that the receptor-cargo complex fully enters the peroxisomal lumen (14–16). Either model is compatible with the existence of the transient PEX5 pore as a plausible model for the translocation step. Therefore, the extent to which the receptor contacts the peroxisomal lumen during import remains unclear.

In recent years it has been shown that the interaction of PEX14 with the N-terminal region of PEX5 affects the cargo binding of PEX5 (17–19). Following the unloading of cargo proteins, the receptor is released from the peroxisome via two interacting groups of proteins: the RING finger peroxins (PEX2, PEX10, and PEX12) and the receptor-release complex consisting of the PEX4-PEX22 subcomplex and the ATPase associated with various cellular activities proteins PEX1, PEX6, and a peroxisomal membrane protein tether, PEX26 in mammals (20), PEX15 in yeast (21), or APEM9 in plants (22). PEX1 and PEX6 form a hexameric complex that has been proposed to thread partially or completely unfolded proteins through a central channel for export (23). Receptors are targeted for release from the importomer by attachment of ubiquitin moieties. To date, four peroxins have been identified as targets of ubiquitination: PEX5, PEX7, PEX18, and PEX20 (21, 24–28). The extent of ubiquitination of the receptor determines its fate. Monoubiquitinated receptors are released and deubiquitinated to begin another import cycle, and polyubiquitinated receptors are targeted for degradation by the 26S proteasome (25, 29, 30). The GTPase rabE1c has also been shown to interact with *Arabidopsis* PEX7 and regulate its turnover (31).

N-terminally truncated mammalian PEX5L lacking the first 110 amino acids displays the same import properties as the full-length peroxin, but the ATP-dependent recycling of this construct is blocked completely. The same is true of PEX5L lacking the first 17 amino acids, implying that the N terminus is not involved in cargo-protein binding or the translocation of the cargo-loaded protein but that it is critical for recycling (32). The N-terminal region of PEX5 has been shown to be the site of ubiquitination, with either monoubiquitination occurring at a conserved cysteine residue (30, 33) or a polyubiquitin chain beginning at either of two conserved lysine residues (34).

PEX1 and PEX6 provide the energetic driving force for the recycling step of the cycle and are the only ATPases known to be associated with the importomer (35). In contrast, the import step is an ATP-independent process (36, 37), instead being driven solely by favorable thermodynamic interactions, first between the cargo and receptor and then between the receptor and the docking complex.

Much of the information that has been garnered on the mechanism of peroxisomal protein import has been through *in vitro* import assays, where isolated peroxisomes or peroxisome containing fractions are incubated with receptor and cargo proteins under varying conditions. Detection in these assays was originally performed through radiolabeling of substrates and radioimaging (38–42). However, these techniques are hindered by the high costs and associated risks of radioactive materials. In recent years, the use of partial proteolysis protection to identify different degrees of membrane protection for various proteins has provided a wealth of knowledge on the fundamentals of import (reviewed in Ref. 43). Studies have determined the requirements for receptors and cargo to reach various stages of protease protection, as identified through the presence, absence, or processing of a protein of interest. Such methods, however, are also limited in their capacity to determine the finer details of interactions within the import process and leave questions unanswered regarding the association of other peroxins with the protein of interest in each stage.

To address the need for tools to study protein interactions in the peroxisomal import cycle, we developed a covalent label transfer strategy for use in *in vitro* import assays. This method allows the demonstration of interaction between PEX5 and PEX14 as purified recombinant protein constructs (PEX5C and PEX14N) and also in *in vitro* peroxisomal import experiments. The PEX5-PEX14 interaction is not dependent on the full N terminus of PEX5 and is the first step in the association of PEX5 with the peroxisomal membrane. We also observed dependence between the ATP driving force of the receptor recycling step and the ability of PEX5 to initially interact with the docking complex at the beginning of the import cycle.

## Experimental Procedures

### 

#### 

##### Recombinant Protein Expression and Purification

pMDH1 (At2g27780) cloned in pET21a between the Nhe1 and Xho1 sites was a gift from Prof. S. Smith (University of Western Australia). Expression and purification were performed as described previously (44). PEX5C and PEX14N were expressed, purified, and detected by immunoblotting as described previously (18).

##### Preparation of Peroxisomes

Sunflower peroxisomes were prepared as described previously (39). The protein concentration of the peroxisomal fraction was determined using the BCA method (Sigma).

##### Preparation of the Crude Cytosolic Fraction

The post-peroxisome supernatant was spun at 100,000 × *g* for 10 min at 4 °C. The supernatant was used as a crude cytosolic fraction.

##### pMDH1 Import Assays

Import assays were performed in a total volume of 200 μl containing (unless stated otherwise) pMDH1 (100 ng), peroxisomal fraction (150 μg), 2 mm ATP, and an ATP-regenerating system comprising 64 mm creatine phosphate and 0.5 mg/ml creatine kinase, cytosolic fraction (135 μg), and PEX5C (15 μg). Organelle buffer B (39) was added to make up volume. Where indicated (see figure legends), import assays were treated with 1 mg/ml thermolysin (Sigma) prior to reisolation through a sucrose cushion. For Triton X-100 treatments, organelles were reisolated, solubilized, and treated with protease.

##### Labeling PEX5C with Sulfo-SBED)

Sulfo-*N*-hydroxysuccinimidyl-2-(6-[biotinamido]-2-(*p*-azido benzamido)-hexanoamido) ethyl-1,3′-dithioproprionate (Sulfo-SBED)^6^ (Thermo Fisher Scientific) was incubated with PEX5C at a molar ratio of 20:1, and the reaction was allowed to proceed for 30 min in the dark at room temperature. Labeled PEX5C was separated from free Sulfo-SBED by size exclusion chromatography. A 1-ml Sephadex G-25 column was washed with 3 column volumes of wash buffer (25 mm HEPES (pH 7.2) and 150 mm NaCl) prior to loading the reaction mixture. Labeled PEX5C was eluted with 100-μl aliquots of wash buffer. The protein content of eluted fractions was detected using the Bradford assay. Fractions containing protein were pooled, and the total protein was concentrated calculated.

##### Label Transfer Import Assay

Label transfer import assays were performed as the pMDH1 import assays described previously in this paper, with the following modifications. At given time points, samples were UV-irradiated at 365 nm for 15 min and reisolated through a sucrose cushion as described previously. The pellets were subsequently washed with wash buffer followed by a carbonate wash (0.1 m Na_2_CO_3_ (pH 11.5)). Each time, the membranes were pelleted at 100000 × *g*. The final pellet was resuspended in wash buffer containing 1× complete EDTA-free protease inhibitors (Roche) and homogenized using a 0.1-ml Dounce homogenizer before loading on an SDS-PAGE gel. For PEX14N competition assays, the PEX14N construct was included in the organelle buffer at the indicated molar ratios.

##### Immunoblotting

SDS-PAGE gels were transferred to nitrocellulose (Thermo), and recombinant pMDH1 and PEX5C were detected using α-His-HRP blotting followed by SuperSignal (Thermo) ECL detection. Biotinylated products were detected using streptavidin HRP conjugate and Upstate Visualizer^TM^ (Merck Millipore).

## Results

### 

#### 

##### PEX5C and pMDH1 Associate with the Peroxisomal Membrane

**FIGURE 1.**
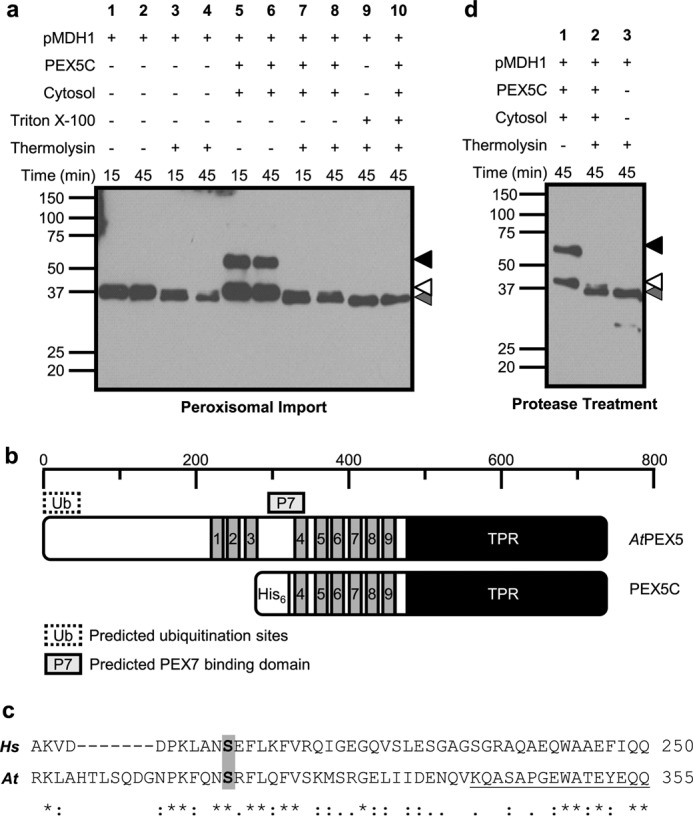
**pMDH1 and PEX5C reisolate with sunflower peroxisomes, but protease treatment is uninformative.** *a*, import reaction in the presence of peroxisomes. Assays were allowed to proceed for the indicated times before reisolation of organelles through a sucrose cushion. The resulting pellets were analyzed by SDS-PAGE and α-His immunoblotting. All assays were performed at 26 °C in the presence of ATP and an ATP-regenerating system. *Black arrowhead*, PEX5C; *white arrowhead*, pMDH1; *grey arrowhead*, pMDH1 degradation product. *b*, schematic of key regions in *Arabidopsis* PEX5 along with the PEX5C construct. *TPR* indicates the PTS1 cargo-binding tetratricopeptide repeat domain. The boxes labeled *1–9* represent the position of the nine W-*X*_3_-F/Y PEX14 binding motifs. The *shaded box* denotes the predicted PEX7 binding domain, and the *dashed box* labeled *Ub* represents the putative ubiquitination sites required for recycling. *c*, ClustalW2 sequence alignment of *Hs*PEX5L and *At*PEX5 amino acid sequences. The key serine residue for PTS2 import mutated in the *Arabidopsis pex5–1* mutant is *shaded*. The *underlined region* denotes the start of the PEX5C construct. *d*, mock import assays performed in the absence of peroxisomes. The *arrowheads* are as described in *a*.

*In vitro* import assays have been applied to study the import of various proteins into isolated mammalian peroxisomes, including PEX5 and thiolase PTS2 cargo (45, 46). In plants, this technique has been applied to the import of several PTS1 cargo proteins and thiolase into peroxisomes (38–42, 47). To further investigate the PTS2 pathway in plants, we attempted to develop an import assay utilizing protease protection analysis of a recombinant PTS2 protein, peroxisomal malate dehydrogenase 1 (pMDH1) from *Arabidopsis thaliana* (At2g22780). *Arabidopsis* pMDH1 is synthesized as a precursor of 37.5 kDa and processed to the mature form by a peroxisomal processing protease, DEG15, that removes the N-terminal 35 amino acids that contain the PTS2 and results in a mobility shift that is easily detected on SDS-PAGE (48, 49). Therefore, it was anticipated that processing would provide a convenient readout for import that could be confirmed by the acquisition of protease resistance by the mature form of the protein. Sunflower peroxisomes were isolated on a Nycodenz step gradient and incubated with recombinant, C terminally hexahistidine-tagged pMDH1 (44) under import conditions, as described under “Experimental Procedures.” The indicated samples were then treated with thermolysin, and the peroxisomes from all import reactions were reisolated on a sucrose cushion and analyzed by SDS-PAGE with α-His-HRP immunoblotting to detect pMDH1 (Fig. 1*a*, *lanes 1–4*). The pMDH1 protein reisolated with peroxisomes after 15 and 45 min of incubation, but there was no evidence of N-terminal processing (Fig. 1*a*, *lanes 1* and *2*). Upon treatment with thermolysin, a small mobility shift of ∼1 kDa was observed upon detailed inspection of the immunoblot (Fig. 1*a*, compare *lanes 3* and *4* with *lanes 1* and *2*), indicating a high degree of resistance to proteolysis. From these results, it was not possible to discern whether the highly limited digestion of pMDH1 by thermolysin was a result of partial translocation across the peroxisome membrane, perhaps because some components required for full translocation were missing in the *in vitro* assay, or because of innate pMDH1 protease resistance.

In a mammalian cell-free import system, inclusion of a PEX5 construct that is unable to bind PTS1 proteins but retains the PEX7 binding domain stimulates thiolase (PTS2 protein) import (46). *Arabidopsis* has only one isoform of PEX5, which corresponds to the mammalian PEX5L and functions as the PTS2 co-receptor (50). Therefore, we hypothesized that the converse situation, providing a recombinant PEX5 protein in excess that could bind PTS1 proteins but not PEX7, could be applied to block import of pMDH1 by competition for the translocon components. The first 454 amino acids of *At*PEX5 are sufficient to recover PTS2 import *in planta* (51). Deletion of the first 340 amino acids of *At*PEX5 (PEX5C) removes the ability of PEX5 to bind to PEX7 and function as a co-receptor but retains five of the diaromatic pentapeptide motifs facilitating interaction with the N terminus of the peroxisomal membrane docking protein PEX14 (Fig. 1, *b* and *c*) (18). Therefore, upon inclusion of PEX5C in import assays, a resultant decrease in protease protection of pMDH1 could be used to infer that the protease protection observed in the absence of PEX5C arises from (partial) import into the peroxisome.

PEX5C was expressed and purified as described previously (18). Import assays were conducted in the presence of PEX5C, which carries an N-terminal His tag, and analyzed by α-His-HRP immunoblotting to detect both pMDH1 and PEX5C as described previously (18) (Fig. 1*a*, *lanes 5–8*). Cytosolic extract from sunflower cotyledons was also included in case some soluble factors such as PEX7, the PTS2 receptor, and PEX5, the co-receptor, were limiting. Fig. 1*a*, *lanes 5* and *6*, shows that both pMDH1 and PEX5C reisolate with peroxisomes to a similar extent after 15 and 45 min of incubation, raising the possibility that pMDH1 may exhibit nonspecific binding to the peroxisome membrane. pMDH1 again showed the same degree of resistance to protease treatment (Fig. 1*a*, compare *lanes 3* and *4* with *lanes 7* and *8*), leading us to question whether the protein was genuinely imported. In contrast, PEX5C was sensitive to externally added protease, resulting in a complete absence of signal (Fig. 1*a*, compare *lanes 5* and *7*, *top band*). Further investigation of the protease sensitivity of pMDH1 by solubilization of peroxisomes with Triton X-100 prior to protease treatment also failed to result in protein degradation (Fig. 1*a*, *lanes 9* and *10*). Comparison of the proteolytic digestion of pMDH1 in the absence of peroxisomes demonstrated the same degree of protease resistance, whereas PEX5C was completely sensitive (Fig. 1*d*). The inherent resistance of pMDH1 to thermolysin in the presence of detergent (Fig. 1*a*) or the absence of peroxisomes (Fig. 1*d*) therefore highlights a problem in the use of protease protection as a measure of import. Not all proteins are amenable, particularly if they adopt a stable folded structure that results in very minor protease digestion that is difficult to resolve by SDS-PAGE, as in the case of pMDH1. Similarly, the complete absence of the PEX5C signal upon protease treatment limits the information that can be garnered about this construct through this method, therefore requiring an alternative method of analysis.

These experiments, however, demonstrated that PEX5C was capable of associating with the peroxisomal membrane and that at least the N terminus bearing the hexahistidine tag was accessible to externally added protease. Because PEX5 is the central multifunctional component of the importomer, we wished to understand more about the nature of the interactions formed by PEX5C. We therefore sought to develop an alternative method for analysis of *in vitro* import assays through a covalent label transfer strategy to allow the determination of interactions formed with PEX5C.

##### Label Transfer Indicates PEX5C Is Monomeric in Solution

**FIGURE 2.**
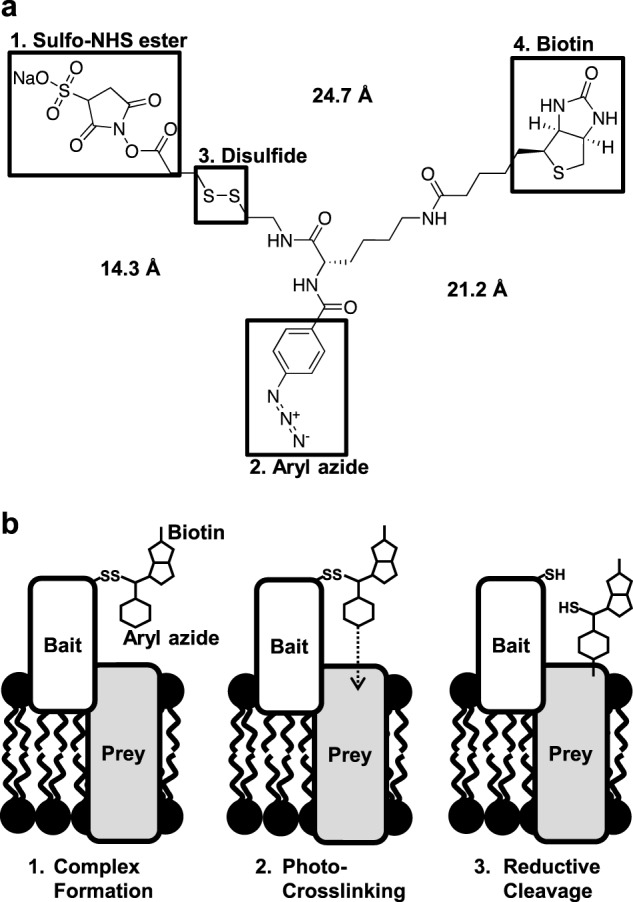
**Structure of the Sulfo-SBED reagent and illustration of the label transfer strategy to detect molecular interactions.** *a*, structure of the Sulfo-SBED cross-linking reagent, containing four main functionalities: the amine reactive Sulfo-NHS ester (*1*) to attach the Sulfo-SBED to free amines in the “bait” protein, an aryl azide (*2*) that can be photoactivated to covalently cross-link to the “prey” protein, a cleavable disulfide (*3*) to remove the linkage to the bait protein, and a biotin tag (*4*) for detection/isolation. Distances between key functional moieties are as provided by the manufacturer (Thermo). *b*, schematic of the cross-linking label transfer strategy to identify molecular interactions. *1*, The bait protein labeled with Sulfo-SBED is incubated with putative binding partners. *2*, UV irradiation forms a covalent linkage to the prey binding partner. *3*, reduction cleaves the disulfide linkage to the bait protein, leaving the biotin label solely attached to the prey protein for detection.

Sulfo-SBED is a tetrafunctional covalent labeling reagent consisting of an amine-reactive sulfomaleimide to allow attachment to bait species, a photoactivatable aryl azide moiety to allow cross-linking to binding partners, a biotin reporter for detection, and a reducible disulfide linkage to allow cleavage from the bait protein and transfer of the biotin moiety to the binding partner (Fig. 2*a*). Biological interactions can be detected through functionalization of the bait protein with the Sulfo-SBED labeling reagent. The functionalized bait protein is then incubated with suspected interaction partners to allow complex formation (Fig. 2*b*, *left panel*). At given time points, the mixture is then UV-irradiated, resulting in photoactivation of the aryl azide to form an aryl nitrene. The aryl nitrene can insert into C-H or N-H bonds or react with nearby nucleophilic residues to form a covalent cross-link between bait and prey species (Fig. 2*b*, *center panel*). Treatment of the cross-linked sample with a reducing agent (*e.g.* DTT in reducing SDS-PAGE sample buffer) cleaves the reducible disulfide link between the bait species and the cross-linking reagent, resulting in the biotin label being covalently attached to only the prey components (Fig. 2*b*, *right panel*). Therefore, this allows the identification of interacting biomolecules by transfer of the biotin label and detection through streptavidin blotting.

**FIGURE 3.**
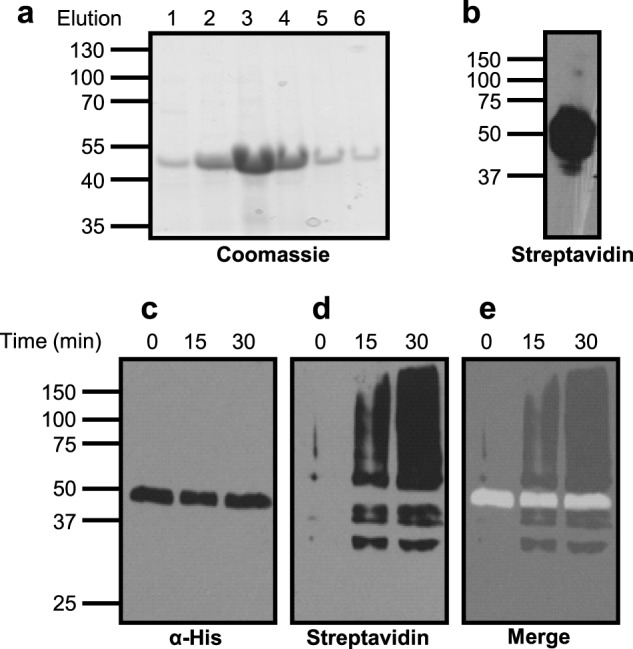
**Labeling of PEX5C with Sulfo-SBED indicates that BED-PEX5C is unable to transfer the biotin label to itself.** Label transfer was performed in buffers as described for the import assays in the absence of peroxisomes. The assays were allowed to proceed for the indicated times prior to UV irradiation. *a*, Coomassie staining of elution fractions 1–6 from PEX5C His tag purification indicates the presence of a low level of residual *E. coli* proteins. *b*, labeling of PEX5C with Sulfo-SBED was performed as outlined under “Experimental Procedures” and confirmed by SDS-PAGE and streptavidin-HRP blotting in the absence of reducing agents (loading equivalent to 50% loading from the label transfer assay). *c*, BED-PEX5C label transfer in the absence of peroxisomes probed with α-His-HRP. *d*, the blot shown in *c* stripped and reprobed with streptavidin-HRP. *e*, the images from *c* and *d* merged, indicating the absence of label transfer to PEX5C.

To determine what, if any, components of the importomer were interacting with PEX5C, purified PEX5C (Fig. 3*a*) was treated with Sulfo-SBED to allow labeling of reactive amine functions (there are 16 Lys residues in the sequence, and reaction may also occur at the N terminus). The resultant PEX5C protein functionalized with (6-[biotinamido]-2-(*p*-azido benzamido)-hexanoamido) ethyl-1,3′-dithioproprionate (BED-PEX5C) was analyzed by SDS-PAGE, followed by streptavidin HRP blotting to confirm labeling (Fig. 3*b*).

Because the full-length PEX5 protein has been shown to oligomerize (52, 53), the ability of the PEX5C construct to self-associate and autolabel was initially investigated under mock import conditions. BED-PEX5C was incubated in import buffer in the absence of peroxisomes and UV-irradiated for 15 min at given time points. The BED cross-linker was cleaved from the bait PEX5C under the reducing conditions of the SDS-PAGE sample buffer, and the samples were separated by SDS-PAGE. Immunoblotting using α-His-HRP detected a band of ∼50 kDa (Fig. 3*c*), consistent with the observed size of PEX5C on SDS-PAGE gels (Fig. 3*a*) (18). Stripping and reprobing with streptavidin-HRP indicated biotinylation of a range of species in solution, most likely corresponding to a low level of residual *Escherichia coli* proteins in the recombinant PEX5C preparation (Fig. 3*d*). Overlaying these two blots indicates that the band detected by the α-His-HRP does not overlap with bands detected by the α-biotin probe (Fig. 3*e*). This demonstrates that the BED-PEX5C construct does not autolabel and is therefore likely to be monomeric in solution, as has been reported previously for the full-length human PEX5 (54).

##### PEX14N Competes with the Peroxisome for Binding to PEX5C

**FIGURE 4.**
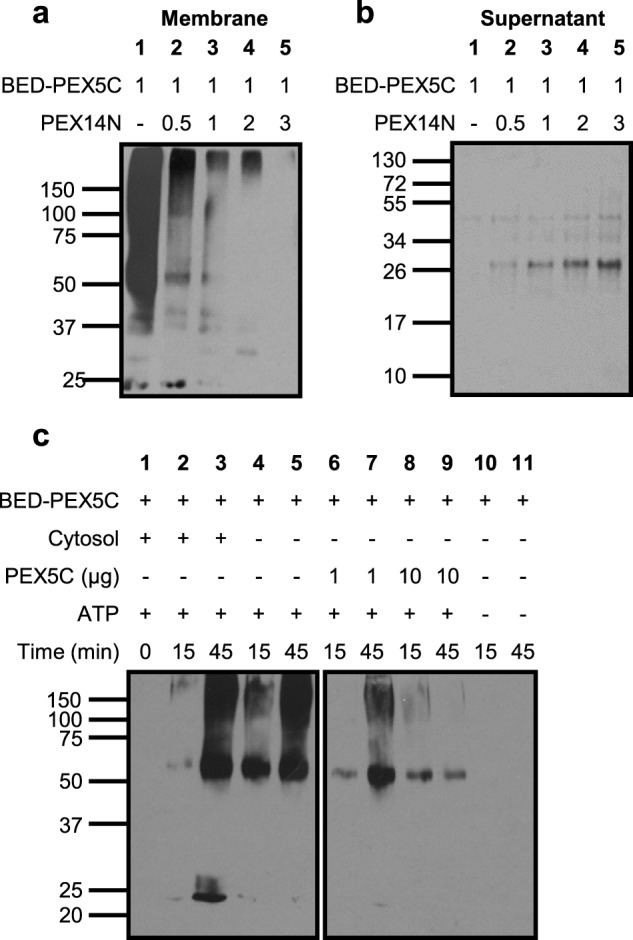
**BED-PEX5C label transfer to peroxisomes can be blocked by increasing concentrations of PEX14N, increasing concentrations of PEX5C, or the absence of ATP but is PTS1 cargo-independent.** Label transfer assays were performed as outlined under “Experimental Procedures” (see Figs. 1 and 2). Reactions were incubated at 26 °C for 45 min and terminated by UV irradiation, and the organelles were reisolated through a sucrose cushion. The membranes were carbonate-washed, and both the pellet and supernatant were analyzed by SDS-PAGE and Western blotting. *a*, import reactions in the presence of increasing concentrations of PEX14N (molar equivalents are shown above the panels). Membrane fractions from import assays were probed with the streptavidin-HRP probe. *b*, equivalent supernatant fractions from *a* probed with the streptavidin-HRP probe. *c*, membrane fraction from import assays using BED-PEX5C investigating the effect of crude cytosolic cargo, unlabeled PEX5C, or the ATP-regenerating system. The panels are from the same exposure of a single blot, with all samples taken from the same experimental repeat.

The BED-PEX5C construct was next employed in import assays to investigate the ability of the construct to interact with the importomer. Import assays were performed as described previously and UV-irradiated for 15 min to facilitate cross-linking. Samples were carbonate-extracted, and the cross-linker was cleaved from BED-PEX5C in reducing SDS-PAGE sample buffer prior to analysis by streptavidin-HRP blotting. Biotin label transfer to the peroxisomal membrane fraction was observed for a range of proteins above ∼50 kDa in mass as well as a band of ∼25 kDa (Fig. 4*a*, *lane 1*).

One expected interaction partner of PEX5C is PEX14 because PEX5C has been shown previously to interact with a PEX14 N-terminal construct in solution (18). Therefore, to verify the specificity of the interacting components at the peroxisomal membrane, cross-linking import assays were conducted in the presence of increasing concentrations of the soluble PEX14N construct (Fig. 4, *a* and *b*). After import and cross-linking, peroxisomes were reisolated, and both the organelle and soluble fractions were analyzed by streptavidin-HRP blotting. At low PEX14N:PEX5C ratios, the majority of the label transfer was to the peroxisomal membrane fraction. As the amount of PEX14N was increased from 0 to 3 equivalents of PEX5C, a decrease in label transfer to the peroxisomal membrane was observed (Fig. 4*a*). At the same time, a concomitant increase in label transfer to the soluble fraction was observed. Label transfer was predominantly to a soluble 26-kDa protein (Fig. 4*b*), corresponding to the correct size for PEX14N on a 15% SDS-PAGE gel (18).

##### BED-PEX5C Labeling of Peroxisomes Requires an ATP Energetic Driving Force

To further assess the applicability of label transfer to peroxisomal import analysis, the temporal requirements of label transfer were investigated. Import assays were performed as described previously, and samples were UV-irradiated to activate labeling at given time points (Fig. 4*c*). In complete import assays, an increase in labeling of peroxisomal membrane proteins over time was observed (Fig. 4*c*, *lanes 1–3*). The omission of cytosol from the import reactions resulted in a higher rate of labeling at the peroxisomal membrane, as evidenced by increased biotinylation after 15 min (Fig. 4*c*, compare *lanes 2* and *4*).

The specificity of the BED-PEX5C interaction with the importomer was investigated through label transfer import experiments in the presence of competing, unlabeled PEX5C. Addition of 1 μg of unlabeled PEX5C to import assays resulted in a marked decrease in label transfer to the peroxisomal membrane, and addition of 10 μg of unlabeled PEX5C resulted in an almost complete loss of biotinylation signal at the membrane (Fig. 4*c*, *lanes 6–9*), demonstrating that the interactions of PEX5C are maintained in the presence of Sulfo-SBED functionalization.

To further investigate the conditions required for interaction of BED-PEX5C with the importomer, label transfer import assays were performed in the presence or absence of the ATP-regenerating system (Fig. 4*c*, *lanes 2* and *3* and 10 and *11*, respectively). Strikingly, the absence of ATP in the import reaction had a pronounced effect, resulting in no label transfer to the peroxisomal membrane being detected (Fig. 4*c*, *lanes 10* and *11*).

## Discussion

For this report, we developed an alternative method for analysis of *in vitro* peroxisomal import through use of a covalent label transfer strategy. Initial attempts to analyze the *in vitro* import of pMDH1 (PTS2 cargo) were hampered by a high degree of resistance to thermolysin treatment, resulting in only minor protease digestion that was hard to resolve by SDS-PAGE, making the determination of import problematic. If pMDH1 peroxisomal protease protection was due to genuine import, then, we reasoned, increased competition from the PTS1 pathway for import would reduce the extent of protease protection. Sequence alignment of the region encoding for PEX7 binding in *Hs*PEX5L indicates its conservation in *At*PEX5 (Fig. 1*c*), and the *Arabidopsis pex5–1* mutant, in which the conserved serine in this region is mutated to leucine, has a specific PTS2 import defect (50). Deletion of the first 340 amino acids of *At*PEX5 (PEX5C) removes the ability of PEX5 to bind to PEX7 and function as a co-receptor, but it retains the ability to bind PTS1 cargo without any decrease in affinity compared with full-length PEX5 (5, 18). A converse strategy has been applied in mammalian peroxisomes where PTS2 import is promoted by sequestering PTS1 cargos with an import-deficient PEX5-tetratricopeptide repeat construct, presumably by alleviating competition at the importomer from PEX5S (non-PTS2 import competent PEX5 variant) loaded with the more abundant PTS1 proteins (46).

Even under such conditions, there was no convincing evidence of pMDH1 import (Fig. 1, *a* and *d*). Such experiments did, however, demonstrate the ability of PEX5C to associate with the peroxisomal membrane. The N-terminal hexahistidine tag of PEX5C was readily cleaved, suggesting that at least this part of the protein is exposed to the cytosol, consistent with reports of full-length PEX5 in mammalian systems (55). This highlighted the intrinsic limitation of such protease protection methods in requiring the existence and identification of an appropriate protease/substrate pair for analysis and led us to employ a non-biased label transfer strategy for analysis.

PEX5C was readily labeled on free lysines using the Sulfo-SBED cross-linking reagent. In the absence of peroxisomes, UV irradiation of the labeled receptor followed by reductive cleavage of the biotin label demonstrated no overlap of biotinylated and polyhistidine-tagged species (Fig. 3*e*), indicating that PEX5C was predominantly monomeric under the assay conditions. This finding is consistent with reports that PEX5 is mainly monomeric at a slightly acidic pH (the import assay was performed at pH 6) and adopts different oligomeric states at more basic pH values (56–58).

Complete label transfer import assays demonstrated a high degree of biotinylation of a range of peroxisomal membrane proteins. Competition experiments in the presence of an N-terminal PEX14 construct demonstrated that this construct could block label transfer from PEX5C to all peroxisomal membrane proteins (Fig. 4, *a* and *b*). This finding is consistent with literature showing that the presence of PEX14 is required for import (59–61). However, crucially, our result demonstrates that it is this interaction between PEX5C and PEX14 that is the first point of interaction between the soluble receptor and the importomer.

The BED-PEX5C construct interacted more strongly with binding partners in the peroxisome membrane in the absence of cytosol, presumably through a reduction in competition from endogenous PEX5. This finding is also consistent with recombinant protein studies that show that PEX5C can interact with the PEX14 N terminus in the absence of PTS1 cargo (18). It has been proposed that binding of PTS1 cargo to PEX5 facilitates rearrangement of the PEX5 N terminus to activate peroxisomal import (62). However, structural studies have shown no significant conformational differences between the cargo-loaded and cargo-free receptors (63). Observations of cargo-free PEX5-PEX14 interactions should, however, be interpreted in the context of the cytosolic environment. Given the high abundance and affinities of PTS1 cargo proteins (5, 64), this will most likely result in the majority of cytosolic PEX5 being in the cargo-loaded state. This means that, although cargo-free receptor-importomer interactions are possible, they will likely be outcompeted by an excess of cargo-loaded receptor. Future biophysical studies will be required to investigate any differences in PEX14 affinity of cargo-loaded or cargo-free PEX5 and to determine the importance of cargo-free interactions.

**FIGURE 5.**
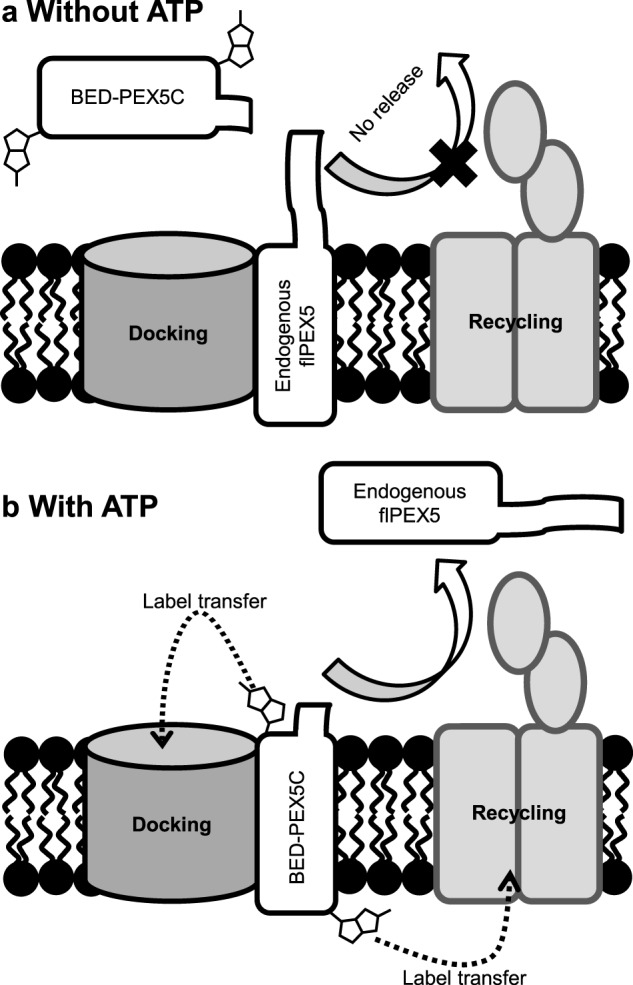
**Model of the export-driven PEX5 import cycle.** *a*, in the absence of ATP to drive receptor release, the import machinery is saturated with endogenous PEX5 receptor, prohibiting any label transfer from BED-PEX5C to the importomer. *b*, ATP drives release of endogenous PEX5 from the import machinery, allowing binding of BED-PEX5 and label transfer to peroxisomal membrane proteins.

In contrast to the ability of PEX5C to interact with the importomer in the absence of cargo, strikingly, labeling of importomer components by PEX5C was shown to be ATP-dependent (Fig. 4*c*). As PEX5C lacks the far N-terminal sites for ubiquitination (Cys^6^, Lys^18^, and Lys^24^ in yeast), it should not be able to be targeted for release from the importomer and, therefore, is not able to participate in the ATP-dependent release step. As demonstrated here, the initial interaction of PEX5C with the importomer is association with PEX14, an interaction that has been predicted to be driven solely by favorable thermodynamic interactions (46), and, indeed, we have previously demonstrated interaction between PEX5C and PEX14N *in vitro* in the absence of ATP (18). Because the absence of an energetic driving force for receptor release prohibits association of the receptor with the docking complex, this suggests a functional connectivity between the docking step and the receptor release step. The isolated peroxisomes presumably already contain PEX5 bound to the importomer (Fig. 5*a*), and, indeed, the conditions for peroxisome isolation (low temperature without ATP) have been reported previously to increase association of PEX5 with the peroxisome membrane (65). Therefore, in our *in vitro* experiments, ATP is most likely required to remove endogenous PEX5 before PEX5C can access the importomer (Fig. 5*b*), and this can explain the observation that *in vitro* import of proteins in the sunflower system is ATP-dependent (38–40, 47). This observation suggests that PEX5 is in contact with importomer components throughout its import cycle rather than being released from the importomer into the peroxisomal lumen and, therefore, supports a simple shuttle model for receptor translocation. The importomer retention of a PEX5 construct possessing a bulky C-terminal tag has been proposed indicate that the release of cargo proteins from the tetratricopeptide repeat domain is a prerequisite for PEX5 export (66), highlighting a potential interdependence of import and export processes. Recent investigations have suggested that mammalian PEX7 is also retained within the importomer throughout its import cycle (67). Retention of PEX5 is consistent with the model proposed by Grou *et al.* (68) and, within this model, would place the cross-linked species at stage 2 (68). This raises the possibility that the rate of cargo import into the peroxisome may be regulated by the rate of ATP-dependent receptor release, as proposed by Schliebs *et al.* (69) in their export-driven import model, rather than the abundance of cargo-loaded receptor available. Indeed, computational modeling supports a cooperatively coupled mechanism where export of one PEX5 molecule from the importomer can only occur with concomitant import of a separate PEX5-cargo complex (70). Further investigations will be required to fully unravel the interplay of factors governing the overall rate of import.

In summary, label transfer provides an alternative means of monitoring *in vitro* import when protease protection methods cannot be applied. Label transfer demonstrated PEX14 as the primary interaction of PEX5C in the import cycle and demonstrated a striking cargo independence and ATP dependence of this interaction. This technique, therefore, affords a broader analytical perspective to protease protection in *in vitro* import, demonstrating the presence or absence of interactions of interest rather than the presence or absence of a protein of interest. Biotinylation of binding partners also affords the opportunity for affinity enrichment and mass spectrometry-based proteomic analysis of the range of potential interactors. Label transfer, therefore, is a powerful tool for analysis of peroxisomal *in vitro* import that affords an enhanced level of understanding of the biochemical mechanism of this process and should be utilized in future studies.

## Author Contributions

M.S.B. prepared pMDH1 and PEX5C and performed the import and label transfer assays. T.L.H. prepared PEX14N and analyzed PEX14N label transfer. K.A.J. developed the label transfer reaction method. T.L.H., S.L.W., and A.B. wrote the paper. All authors designed the experiments, analyzed the results, and approved the final version of the manuscript.

## Footnotes

6 The abbreviations used are:

Sulfo-SBED: sulfo-*N*-hydroxysuccinimidyl-2-(6-[biotinamido]-2-(*p*-azido benzamido)-hexanoamido) ethyl-1,3′-dithioproprionate
BED: (6-[biotinamido]-2-(*p*-azido benzamido)-hexanoamido) ethyl-1,3′-dithioproprionate.
